# Supplementary material for: Resolving the full spectrum of human genome variation using Linked-Reads
Source: Genome Res. 2019 Apr;29(4):635–45. doi: 10.1101/gr.234443.118 (PMC6442396; doi:10.1101/gr.234443.118)
Supplement: Supplemental Material [file supp_gr.234443.118_Supplemental_longranger-2.2.zip › longranger-2.2.tar.bz2!/lr2.2-public/lib/stan/cmdstan/stan/doc/api/html/namespacestan_1_1io.html]

Stan: stan::io Namespace Reference


|  |  |
| --- | --- |
|  | Stan  2.5.0  probability, sampling & optimization |


- Main Page
- Related Pages
- Namespaces
- Classes
- Files

- Namespace List
- Namespace Members

All Classes Namespaces Files Functions Variables Typedefs Enumerations Enumerator Friends Macros Pages

- stan
- io

Classes |
Functions

stan::io Namespace Reference

Input/output utilities.
More...

|  |  |
| --- | --- |
| Classes | |
| class | cmd\_line |
|  | Parses and stores command-line arguments. More... |
|  | |
| class | csv\_writer |
|  | Writes Stan variables in comma-separated-value format to an output stream. More... |
|  | |
| class | dump\_writer |
|  | Writes data into the S-plus dump format. More... |
|  | |
| class | dump\_reader |
|  | Reads data from S-plus dump format. More... |
|  | |
| class | dump |
|  | Represents named arrays with dimensions. More... |
|  | |
| class | mcmc\_writer |
|  | mcmc\_writer writes out headers and samples More... |
|  | |
| class | reader |
|  | A stream-based reader for integer, scalar, vector, matrix and array data types, with Jacobian calculations. More... |
|  | |
| struct | stan\_csv\_metadata |
|  | |
| struct | stan\_csv\_adaptation |
|  | |
| struct | stan\_csv\_timing |
|  | |
| struct | stan\_csv |
|  | |
| class | stan\_csv\_reader |
|  | Reads from a Stan output csv file. More... |
|  | |
| class | var\_context |
|  | A `var_reader` reads array variables of integer and floating point type by name and dimension. More... |
|  | |
| class | writer |
|  | A stream-based writer for integer, scalar, vector, matrix and array data types, which transforms from constrained to a sequence of constrained variables. More... |
|  | |

|  |  |
| --- | --- |
| Functions | |
| void | pad\_help\_option (std::ostream \*o, const std::string &option="", unsigned int width=20) |
|  | Print help option with padding. More... |
|  | |
| void | print\_help\_helper (std::ostream \*o, const std::string &key\_val, const std::string &msg, const std::string &note="") |
|  | Prints single print option to output ptr if non-null. More... |
|  | |
| void | print\_help\_option (std::ostream \*o, const std::string &key, const std::string &value\_type, const std::string &msg, const std::string &note="") |
|  | Prints single print option to output ptr if non-null. More... |
|  | |
| template<> | |
| bool | cmd\_line::bare< std::string > (size\_t n, std::string &x) const |
|  | |
| template<> | |
| bool | cmd\_line::val< std::string > (const std::string &key, std::string &x) const |
|  | |
| std::string | utc\_time\_string () |
|  | Return the current coordinated universal time (UTC) as a string. More... |
|  | |

## Detailed Description

Input/output utilities.

## Function Documentation

template<>

|  |  |  |  |
| --- | --- | --- | --- |
| bool stan::io::cmd\_line::bare< std::string > | ( | size\_t | *n*, |
|  |  | std::string & | *x* |
|  | ) |  | const |

Definition at line 274 of file cmd\_line.hpp.

template<>

|  |  |  |  |
| --- | --- | --- | --- |
| bool stan::io::cmd\_line::val< std::string > | ( | const std::string & | *key*, |
|  |  | std::string & | *x* |
|  | ) |  | const |

Definition at line 284 of file cmd\_line.hpp.

|  |  |  |  |
| --- | --- | --- | --- |
| void stan::io::pad\_help\_option | ( | std::ostream \* | *o*, |
|  |  | const std::string & | *option* = `""`, |
|  |  | unsigned int | *width* = `20` |
|  | ) |  |  |

Print help option with padding.

Print 2 spaces, the specified help option, then pad to the specified width with spaces. If there is not room for at least 2 padding spaces, start a new line and pad out to width.

Parameters
:   |  |  |
    | --- | --- |
    | option | Option to print (default to empty string). |
    | width | Width of option (defaults to 20). |
    | o | Output stream ptr, default to null |

Definition at line 27 of file cmd\_line.hpp.

|  |  |  |  |
| --- | --- | --- | --- |
| void stan::io::print\_help\_helper | ( | std::ostream \* | *o*, |
|  |  | const std::string & | *key\_val*, |
|  |  | const std::string & | *msg*, |
|  |  | const std::string & | *note* = `""` |
|  | ) |  |  |

Prints single print option to output ptr if non-null.

Parameters
:   |  |  |
    | --- | --- |
    | o |  |
    | key\_val |  |
    | msg |  |
    | note |  |

Definition at line 49 of file cmd\_line.hpp.

|  |  |  |  |
| --- | --- | --- | --- |
| void stan::io::print\_help\_option | ( | std::ostream \* | *o*, |
|  |  | const std::string & | *key*, |
|  |  | const std::string & | *value\_type*, |
|  |  | const std::string & | *msg*, |
|  |  | const std::string & | *note* = `""` |
|  | ) |  |  |

Prints single print option to output ptr if non-null.

Parameters
:   |  |  |
    | --- | --- |
    | o |  |
    | key |  |
    | value\_type |  |
    | msg |  |
    | note |  |

Definition at line 74 of file cmd\_line.hpp.

|  |  |  |  |  |
| --- | --- | --- | --- | --- |
| std::string stan::io::utc\_time\_string | ( |  | ) |  |

Return the current coordinated universal time (UTC) as a string.

Output is of the form "Fri Feb 24 21:15:36 2012"

Returns
:   String representation of current UTC.

Definition at line 18 of file util.hpp.


---

[ Stan Home Page ]

*© 2011–2014,
Stan Development Team.*
